# RETRACTION: Horizontal Necklines Correction With Absorbable Braided Polydioxanone Threads: Case Series

**DOI:** 10.1111/srt.70186

**Published:** 2025-06-05

**RETRACTION**: K.‐H. Yi, S.‐B. Kim, D. Chua, M. B. Stefanelli, M. Alfertshofer, and V. Vachiramon, “Horizontal Necklines Correction With Absorbable Braided Polydioxanone Threads: Case Series,” *Skin Research and Technology* 30, no. 3 (2024): e13617, https://doi.org/10.1111/srt.13617.

The above article, published online on 5 March 2024 in Wiley Online Library (wileyonlinelibrary.com), has been retracted by agreement between *Skin Research and Technology*; and John Wiley & Sons Ltd. The article has been accepted for publication following an inadequate peer review process. Following publication of the article, concerns were identified regarding the reliability of the data presented, as several statements within the article were found to be discrepant and/or overstated. Additionally, inconsistencies in Figures 2 and 4 were identified. The material provided by the authors upon request was insufficient to address the concerns. As a result, the article is retracted due to the editors' loss of trust in the reliability of the presented results. The authors have been notified of the retraction decision.

